# Optimizing radiation dose and image quality in neonatal mobile radiography

**DOI:** 10.1093/rpd/ncaf080

**Published:** 2025-07-18

**Authors:** Takahiko Maeda, Makoto Hara, Hiroyuki Yamasaki, Makoto Nakahara, Yoshinori Tanabe

**Affiliations:** Department of Radiology, Hyogo Prefectural Kobe Children’s Hospital, 1-6-7, Minami, Kobe, Hygo 650-0047, Japan; Graduate School of Health Sciences, Department of Radiological Technology, Okayama University, 2-5-1, Shikata, Kita, Okayama 700-8525, Japan; Department of Radiology, Hyogo Prefectural Kobe Children’s Hospital, 1-6-7, Minami, Kobe, Hygo 650-0047, Japan; Department of Radiology, Hyogo Prefectural Kobe Children’s Hospital, 1-6-7, Minami, Kobe, Hygo 650-0047, Japan; Department of Radiology, Hyogo Prefectural Tamba Medical Center, Hikami, 2002-7, Tamba, Hyogo 669-3495, Japan; Faculty of Medicine, Graduate School of Health Sciences, Okayama University, 2-5-1, Shikata, Kita, Okayama 700-8525, Japan

## Abstract

Children are more susceptible to radiation exposure than adults. Therefore, determining an appropriate radiation dose requires balancing and minimizing radiation exposure while maintaining image quality (IQ) for accurate diagnosis. We evaluated the optimal radiation dose parameters for neonatal chest and abdominal mobile radiography by assessing entrance surface dose and IQ indices. A range of exposure parameters was tested on neonatal and acrylic phantoms, and the optimal settings were determined through visual and physical evaluations. Overall, 65 kVp and 1.2 mAs provided the best balance between minimizing radiation exposure and maintaining high IQ for neonates. This study offers essential insights into optimizing radiographic conditions for neonatal care, contributing to safe and effective radiological practices. These optimized parameters can help guide future clinical applications by ensuring reduced radiation risk and enhanced diagnostic accuracy.

## Introduction

Children are more susceptible to radiation exposure than are adults. Therefore, optimizing radiation doses in children requires special effort. The goal is to reduce radiation exposure while maintaining the image quality (IQ) required for diagnosis [1]. New diagnostic radiology equipment is constantly being developed to reduce radiation exposure while maintaining image quality, and facilities must determine the appropriate imaging conditions based on the characteristics of the equipment [2, 3].

Initiatives such as the Image Gently campaign aim to reduce radiation doses while maintaining diagnostic quality [2]. Automatic exposure control, a method for optimizing radiation dose, is frequently used in adult imaging. However, in smaller body sizes, such as newborns, overdose, or underdose may occur due to the positioning of multiple sensors relative to the organs [4, 5]. Therefore, we considered optimizing the radiation doses for pediatric radiological imaging at each facility according to age and body size from newborns to ~10-year-olds [5, 6].

Mobile radiography is a common examination in pediatric radiology [7]. For newborns with respiratory disorders or circulatory symptoms, mobile radiography can be performed in a minimally invasive manner in incubators while maintaining appropriate temperature and humidity [8]. This technique is used to assess the lungs, pulmonary blood vessels, heart, bones, gas levels, and catheters.

Radiographic imaging equipment has transitioned from film screens to computer radiography and flat-panel detectors, significantly improving the radiographic sensitivity of the detectors and reducing radiation exposure [9]. Imaging systems are continuously being developed due to the efforts of radiation imaging diagnostic manufacturers. Evaluating the characteristics and IQ of new imaging systems before starting clinical trials and capturing images at appropriate radiation doses is crucial [4, 5, 10].

Accurate diagnosis and determination of the appropriate radiation dose are important for evaluating IQ and reducing exposure [11]. Image evaluations are widely performed using physical index assessments and visual evaluations. The risk of cancer varies depending on the age of the child. Therefore, determining the exposure parameters according to the child’s body size is important. The International Commission on Radiological Protection guidelines have also been used to evaluate effective doses and examine diagnostic reference levels, considering the age of the standard pediatric phantom [12]. Radiation-imaging diagnostic systems differ in every facility. Hence, it is necessary to determine the optimal exposure parameters for each facility.

Notably, some studies have conducted physical evaluations of low-contrast resolution and visual IQ assessments using the CDRAD phantom to determine the imaging exposure parameters [13]. However, to the best of our knowledge, no study has examined methods for determining exposure parameters specifically for mobile pediatric radiography using radiation exposure doses and visual IQ evaluations. Therefore, we aimed to determine the optimal exposure parameters for mobile neonatal chest and abdominal radiography by examining entrance surface doses, visual evaluations, and physical index assessments. We present our findings and hope that this study will help determine the exposure parameters for mobile pediatric radiography.

## Methods

### Equipment and analysis system

**Figure 1:**
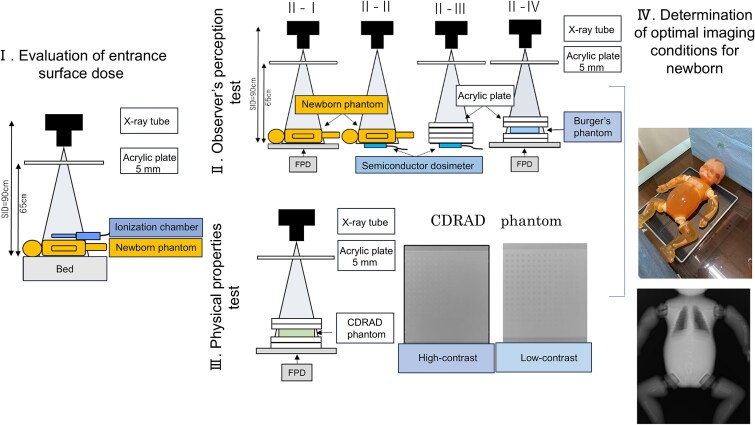
Flow chart of the study. I. Evaluation of the entrance surface dose using an ionization chamber and a neonatal phantom; II. Observer’s perception test using neonatal and Burger’s phantoms; III. Physical properties test using CDRAD phantom; IV. Determination of optimal imaging conditions using neonatal phantoms.

A mobile radiography machine, Sirius130HP (Hitachi, Tokyo, Japan), and an indirect-conversion flat-panel detector device, CALNEO Smart C47 (Fujifilm, Tokyo, Japan), were used. We evaluated the incident surface dose using a semiconductor dosemeter (Unfors Xi, TOREC), an ionization chamber dosemeter (Radcal9015 6 cc chamber, Radcal Corporation), a neonatal phantom (S/N 16C-17, Kyoto Scientific), a concave-convex Burger phantom (Kyoto Scientific), an acrylic plate (400 × 400 × 10 mm), and a CDRAD2.0 phantom (Artinis Medical Systems). Visual evaluation was performed using a medical liquid crystal display (LCD) monitor (EIZO Radiforce MX215, EIZO Co., Ishikawa, Japan) with a resolution of 1600 × 1200 pixels and a contrast ratio of 1500:1. Image analysis was performed using the CDRAD Analysis software (Artinis Medical System, The Netherlands) and Synapse Vincent Ver.6.7.0007 (Fujifilm, Tokyo, Japan). The verification parameters were determined based on the entrance surface dose (Fig. 1). The optimal exposure parameters for chest and abdominal radiography of newborns were determined by visual and physical evaluations.

### Evaluation of the entrance surface dose

The dose on the body surface of the neonatal phantom was measured using an ionization chamber dosemeter with a mobile radiography device. In addition, because imaging was assumed to take place in an incubator, the distance between the X-ray tube focus and X-ray detector was set to 90 cm, and the distance to the top of the incubator was set to 65 cm (Fig. 1). Exposure parameters were adjusted according to body mass: tube voltages of 50, 52, 50, 50, and 52 kV, and tube current-time products of 1.6, 2.0, 2.5, and 2.5 mAs for body weights of 1.0, 1.5, 2.0, 2.5, and 3.0 kg. The entrance surface dose for the 3.0 kg exposure parameters (52 kV, 2.5 mAs) used in the conventional system served as the reference value, and comparisons were made with other exposure parameters.

### Observer’s perception test

#### Contrast adjustment

Imaging was performed using a neonatal phantom under the same exposure parameters as those used in Section: Evaluation of the entrance surface dose, assuming imaging in a couplabase. The sensitivity (S) and latitude (L) values ​​were set such that the contrast remained constant for each exposure parameter. Here, the contrast was adjusted to distinguish the mediastinum, lung field, and abdomen.

#### Evaluation of radiation dose to the neonatal phantom using a semiconductor dosemeter

A semiconductor dosemeter was placed on the back of the neonatal phantom, and the dose was measured under the same exposure parameters as in Section: Evaluation of the entrance surface dose. Here, the dosemeter was also placed on the spine and heart to increase measurement points and improve the reliability of the observer’s perception test.

#### Comparative dosimetry study using acrylic plates of varying thicknesses

The observer’s perception test was performed using the acrylic plate, and the thickness of the acrylic plate was adjusted to match that of the neonatal phantom. Doses were measured using a semiconductor dosemeter under the same exposure parameters as in Section: Evaluation of the entrance surface dose, and the acrylic plate thickness was determined to be equivalent to the value calculated using Section:Evaluation of radiation dose to the neonatal phantom using a semiconductor dosemeter.

Using the acrylic plate thickness derived in Section: Comparative dosimetry study using acrylic plates of varying thicknesses, the uneven Burger phantom was placed at the center to evaluate the visibility of the subject’s center. Images were captured using the exposure parameters listed in Section: Evaluation of the entrance surface dose. As in Section:Evaluation of radiation dose to the neonatal phantom using a semiconductor dosemeter, the spine and heart were assumed, and an observer’s perception test was performed on four patterns of the uneven Burger phantoms. The samples were observed using a medical LCD monitor, and the contrast was adjusted using the S and L values from Section: Contrast adjustment. The observer was free to move and enlarge the images. Observations were independently performed by 10 clinical radiologists with 5–30 years of experience, who ranked the samples based on their visual clarity. The evaluation was conducted using the rank-based normalization method.

### Physical evaluation

#### Quantitative evaluation of CDRAD2.0 phantom IQ using IQF analysis

The CDRAD2.0 phantom was imaged using the exposure parameters outlined in Section: Evaluation of the entrance surface dose. The IQ figure (IQF) was calculated using CDRAD Analysis. Two analysis patterns for the spine and heart were conducted, as described in Section: Evaluation of radiation dose to the neonatal phantom using a semiconductor dosemeter. The IQF_inv_ values were determined using the following equation [14, 15]:

${IQF}_{inv}=\frac{100}{\sum_{i-1}^{15}{C}_i\cdot{D}_{i, th}}$ (1)

Here, IQF is calculated as the sum of the visible diameter (Di,th) and the product of depth (Ci) across all 15 columns. The variable Di,th denotes the smallest diameter in column (i), which contains a correctly detected visible hole, whereas Ci represents the contrast of the object in column (i).

#### Contrast-noise ratio analysis of spine and heart patterns in CDRAD2.0 phantom images

Using the CDRAD2.0 phantom images obtained in Section: Quantitative evaluation of CDRAD2.0 phantom IQ using IQF analysis, the contrast-noise ratio (CNR) for the two patterns for the spine and heart was calculated using the VINCENT software, as described in Section: Evaluation of radiation dose to the neonatal phantom using a semiconductor dosemeter.

### Statistical analysis

Rank-based normalization was analysed using free software (Central Department of Radiology, Nara Medical University Hospital, Japan) provided by Nakamae [16]. Pearson correlation coefficient (r) was analysed using the Statistical Package for Social Sciences software version 29 (IBM Corporation, Armonk, NY, USA). A two-tailed test (*P*-value) was used for statistical significance testing (*P* < 0.01).

## Results

**Figure 2:**
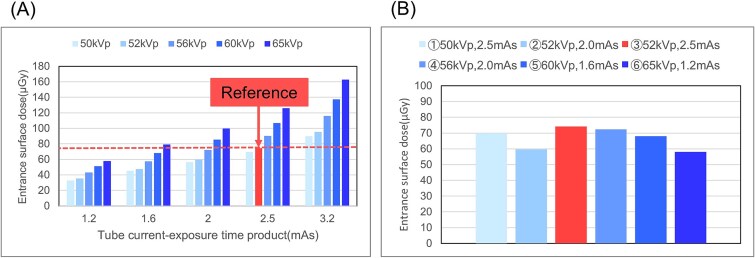
(A) Entrance surface dose and (B) exposure parameter results.

Figure 2 shows the entrance surface dose results. The entrance surface dose for the 3.0 kg exposure parameters (52 kV, 2.5 mAs) served as the reference value (Fig. 2(A)). The exposure parameters corresponding to a lower entrance surface dose were determined progressively for each tube voltage: ① 50 kV, 2.5 mAs; ② 52 kV, 2.0 mAs; ③ 52 kV, 2.5 mAs (reference value); ④ 56 kV, 2.0 mAs; ⑤ 60 kV, 1.6 mAs; and ⑥ 65 kV, 1.2 mAs (Fig. 2B).

**Figure 3:**
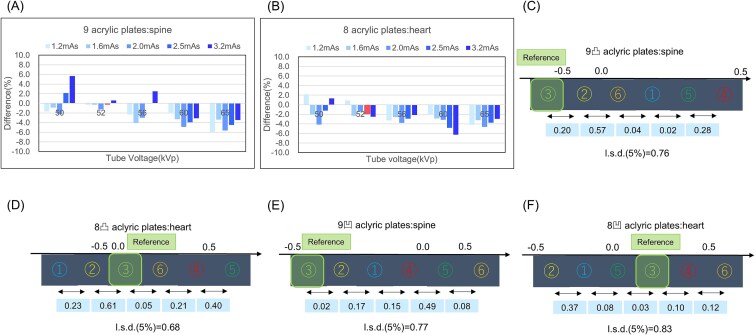
Results of dose difference between the acrylic plates and the neonatal phantom; (A) nine acrylic plates and spine, (B) eight acrylic plates and heart, the rank-based normalization method; contrast: (C) nine acrylic plates, (D) eight acrylic plates, spatial resolution: (E) nine acrylic plates, (F) eight acrylic plates.

The dose difference between the spine and heart in the neonatal phantom and acrylic plates was calculated. Since the maximum difference was small (~6%), the spine and heart doses measured using nine and eight acrylic plates, respectively, were comparable to those of the neonatal phantom (Fig. 3A and B). The observer’s perception test was conducted using the specified exposure parameters, and the distance was calculated using a rank-based normalization method.

In the contrast-detail evaluation of the CDRAD2.0 phantom, the spine ranking with nine acrylic plates was: ④ (mean, 0.49) > ⑤ (0.21) > ① (0.19) > ⑥ (0.15) > ② (−0.42) > ③ (SD, −0.62). When the least significant difference (LSD) was set to 5% (LSD = 0.76), a statistically significant difference was observed for all comparisons, except between ② and ③(Fig. 3C). For the heart with eight acrylic plates, the ranking was ⑤ (0.74) > ④ (0.34) > ⑥ (0.13) > ③ (0.08) > ② (−0.53) > ① (−0.76), with an LSD of 0.68 (5%), showing that only ① differed significantly from ③(Fig. 3D).

In the spatial resolution evaluation of the CDRAD2.0 phantom, the ranking for the spine with nine acrylic plates was ⑥ (0.53) > ⑤ (0.45) > ④ (−0.04) > ① (−0.19) > ② (−0.36) > ③ (−0.38), with an LSD of 0.77 (5%), indicating that ⑤ and ⑥ were statistically different from ③(Fig. 3E). The ranking for the heart with eight acrylic plates was ⑥ (0.53) > ④ (0.45) > ③ (−0.04) > ⑤ (−0.19) > ① (−0.36) > ② (−0.38), with an LSD of 0.83 (5%), indicating no statistically significant difference from ③(Fig. 3F).

The overall rankings from the observers’ perception tests are shown in Table 1.

**Table 1:** Results of the rank-based normalization method using the CDRAD2.0 phantom.

Exposure parameters	Contrast detail		Spatial resolution		Total
	9 plates	8 plates	9 plates	8 plates	18
①	3	6	4	5	18
②	5	5	5	6	21
③	6	4	6	3	19
④	1	2	3	2	8
⑤	2	1	2	4	9
⑥	4	3	1	1	9

The rankings of ④ to ⑥ were better than those of ① to ③ (Table 1). Results showing a significant difference for ① (of 8 convexities) were excluded from verification because visibility was lower than that of the standard exposure parameters.

**Figure 4:**
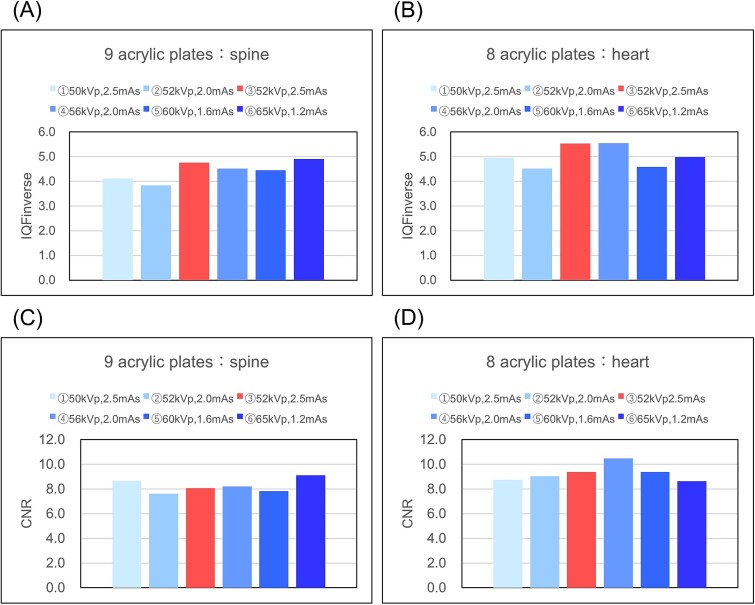
Results of the physical properties test: IQFinverse; (A) spine, (B) heart, contrast-noise ratio (CNR); (C) spine, (D) heart.

The calculated IQFinv and CNR values are shown in Fig. 4. The IQFinv was ① 4.12, ② 3.83, ③ 4.76, ④ 4.52, ⑤ 4.45, and ⑥ 4.90 for the spine, and ① 4.95, ② 4.52, ③ 5.53, ④ 5.55, ⑤ 4.58, and ⑥ 4.98 for the heart, with ⑥ being the higher value for the spine and ④ being the higher value for the heart(Fig. 4(A),(B)).

The CNR was ① 8.67, ② 7.61, ③ 8.06, ④ 8.20, ⑤ 7.83, and ⑥ 9.10 for the spine, and ① 8.73, ② 9.03, ③ 9.37, ④ 10.47, ⑤ 9.39, and ⑥ 8.63 for the heart, with ⑥ being the highest value for the spine and ④ being the highest value for the heart(Fig. 4C and D).

**Figure 5:**
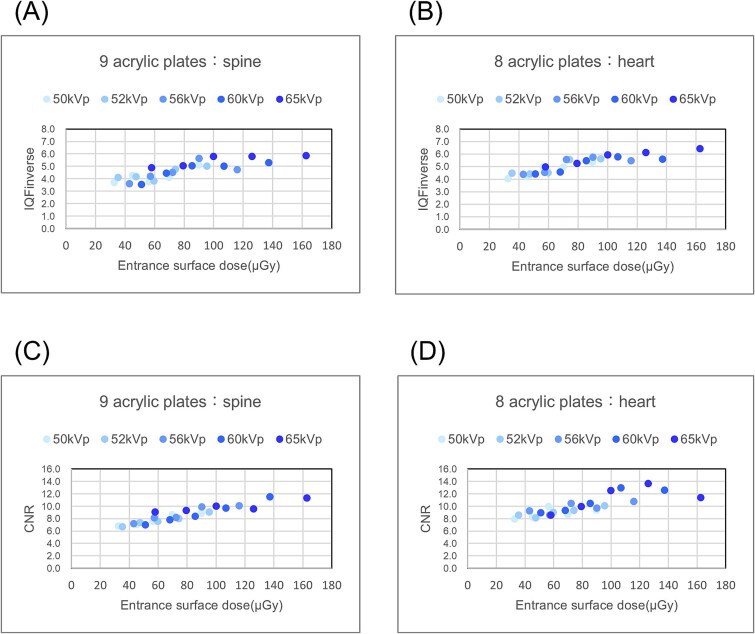
Relationship between the incident surface dose and physical parameters. IQF inverse: (A) spine (r:0.83, *P* < 0.001), (B) heart (r:0.89, *P* < 0.001). Contrast-noise ratio (CNR): (C) spine (r:0.93, *P* < 0.001), (D) heart (r:0.82, *P* < 0.001). Statistical significance (*P* < 0.01) was determined using Pearson’s correlation coefficient (r) and a two-tailed test.

To confirm the reliability, Fig. 5 shows the correlation between the incident surface dose measured and the physical index evaluation (Fig. 5A-D). A strong correlation was observed between the physical index evaluation and the incident surface dose for both indices, ensuring reliability.

Table 2 shows the characteristics of the verified evaluation indices.

**Table 2:** Characteristics of the evaluation index.

Evaluation items	Characteristics
Observer’s perception	④ to ⑥ have high visibility (④ is the highest)
Physical properties	IQF_inv_ and CNR were high in the spine (⑥) and in the heart (④).
Incident Surface Dose	At an incident surface dose lower than standard ③, the difference between maximum dose ④ and minimum dose ⑥ is 14.3 μGy.

The results for ④ and ⑥ were equivalent under the exposure parameters used for verification. Therefore, 65 kV and 1.2 mAs (parameter ⑥), which provide a lower entrance surface dose, were determined to be the optimal settings for chest and abdominal imaging in newborns (3.0 kg).

## Discussion

In this study, the observer’s perception test, entrance surface dose, and physical index evaluations were performed to identify the optimal exposure parameters for mobile neonatal chest and abdominal radiography.

As technology advances, optimizing the IQ and radiation dose for neonatal chest imaging is essential when new devices are introduced [17]. Uncertainty exists in the output of X-ray devices, with previous studies indicating that the discrepancy between the displayed and measured values of the incident surface dose is <20% [18]. As demonstrated in this study, evaluating the characteristics of newly introduced systems in advance is crucial, as we verified the exposure parameters and entrance surface dose in the mobile X-ray system. Objective evaluation of the X-ray devices at each facility to determine optimal imaging conditions will lead to a reduction in the Diagnostic Reference Level (DRL) based on percentiles and improve radiation protection for children [19, 20].

We used exposure doses to compare the images with the appropriate dose for each tube voltage based on the previous parameters used for comparative evaluation at our facility. The optimal exposure parameters for infants and young children are not available in published guidelines, which typically reference the 75th percentile applicable to any system. Therefore, we concluded that these values may not be suitable for newer systems with improved detector sensitivity [5]. In addition, by examining the exposure parameters based on the exposure dose rather than the technical settings, we provided reference exposure parameters that eliminated the uncertainty of output doses, which differed among other facilities.

Radiation exposure should be minimized in children due to their increased sensitivity to ionizing radiation, and achieving contrast between adjacent structures is equally important [21]. Evaluations of the neonatal spine and heart phantoms using acrylic plates showed optimal results for the spine at ④ (56 kV, 2.5 mAs) and ⑤ (60 kV, 1.6 mAs) and for the heart at ⑥ (65 kV, 1.2 mAs). Even in infants, anatomical structures vary depending on the imaging site, and X-ray devices allow for multiple tube voltage settings. Therefore, slight adjustments to the tube voltage can enhance contrast and reduce radiation exposure.

During the physical evaluation, IQFinverse and CNR were elevated at ⑥ (65 kV, 1.2 mAs) for the spine and ④ (56 kV, 2.5 mAs) for the heart. Objective physical indicators are also essential for determining the optimal exposure parameters, and these indicators can serve as a reference for the final decision.

The American Society of Radiologic Technologists guidelines recommend using the highest tube voltage within the appropriate range and lowering the mA value to maintain a consistent dose at the image receptor to determine the optimal exposure parameters [22, 23]. Moreover, previous studies have reported that a tube voltage of 60–65 kV is optimal for chest radiography in newborns. Consequently, we established the optimal exposure parameters for chest and abdominal radiography in newborns (3.0 kg) to be 65 kV and 1.2 mAs, as calculated in this study.

This study had some limitations. We did not account for the material or distance of the base. However, because materials vary across facilities, we believe that the method used in this study can still assist in determining the optimal exposure parameters. Moreover, we did not evaluate actual clinical images. However, the objective evaluation of the method in this study can serve as base data for the periodic evaluation of equipment deterioration and system updates. The primary purpose of the CDRAD phantom is quality control; however, it can be useful for evaluating the optimization of clinical IQ with limited tools. In the future, artificial intelligence technology and Monte Carlo simulations can be used to develop periodic evaluation methods based on clinical images and evaluation methods that include organ doses, which could lead to timely and high-quality evaluation methods [24, 25]. Furthermore, in future studies, we plan to determine the optimal exposure parameters for premature infants, who are more frequently radiographed.

## Conclusion

We determined the optimal exposure parameters for neonatal chest and abdominal mobile radiography using a neonatal phantom and various evaluation indices. In this study, the optimal exposure parameters for chest and abdominal radiography in newborns were (3.0 kg) at 65 kV and 1.2 mAs. The incident surface dose between the displayed and measured values did not match completely, and we believe that by using this research method to determine the optimal conditions at each facility, the DRL can be reduced based on percentiles and improve radiation protection for children.

## Data Availability

Data supporting the findings of this study are available from the corresponding author upon reasonable request.
